# Associations between leukocyte count and lipid-related indices: Effect of age and confounding by habits of smoking and alcohol drinking

**DOI:** 10.1371/journal.pone.0281185

**Published:** 2023-01-31

**Authors:** Ichiro Wakabayashi

**Affiliations:** Department of Environmental and Preventive Medicine, School of Medicine, Hyogo Medical University, Nishinomiya, Hyogo, Japan; University of Chicago, UNITED STATES

## Abstract

Leukocyte count in peripheral blood is an acute-phase reactant and is associated with the risk of atherosclerotic diseases. Blood lipid profile, a major risk factor of cardiovascular disease, is known to be associated with leukocyte count, but it remains to be determined how this association is affected by other factors including lifestyle and age. The subjects were 11261 Japanese middle-aged men (30~65 years old) who had received health checkup examinations. The relationships of leukocyte count with lipid-related indices (ratio of LDL cholesterol to HDL cholesterol [LDL-C/HDL-C], ratio of triglycerides to HDL cholesterol [TG/HDL-C] and cardiometabolic index [CMI]) were investigated. Leukocyte count, LDL-C/HDL-C, TG/HDL-C and CMI were significantly higher in smokers than in nonsmokers, while leukocyte count and LDL-C/HDL-C were significantly lower in regular drinkers than in nondrinkers. Both in overall subjects and subjects without habits of smoking and drinking, LDL-C/HDL-C, TG/HDL-C and CMI were significantly higher in the 2nd and 3rd tertiles for leukocyte count than in the 1st tertile and tended to be higher with an increase of the tertile. Odds ratios for high TG/HDL-C and high CMI of the 3rd vs. 1st tertiles for leukocyte count tended to be lower with an increase of age, and odds ratios for high TG/HDL-C and high CMI of the interaction term, consisting of age (60~65 vs. 30~39 years) and tertile (3rd vs. 1st tertiles for leukocyte count), were significantly lower than the reference level. In conclusion, leukocyte count is associated with lipid-related indices, and the associations are independent of smoking and alcohol drinking and tend to be weaker with an increase of age in Japanese middle-aged men.

## Introduction

Leukocyte count in peripheral blood, a marker of inflammation, is known to be a predictor of cardiovascular events [1]. Blood lipid profile, a major risk factor of cardiovascular disease, is also related to leukocyte count: leukocyte count showed positive associations with total cholesterol, LDL cholesterol and triglycerides and a negative association with HDL cholesterol [2, 3]. These associations are mediated by the chronic inflammatory process of arterial walls in progression of atherosclerosis as the common pathogenesis of cardiovascular disease. In addition, hypercholesterolemia induces proliferation of hematopoietic stem cell progenitors in mice [4], though there is little evidence currently that demonstrates a similar process in humans and thus further studies are needed to confirm this hypothesis.

Lifestyles including diet, nutrition, physical activity and habits of smoking and alcohol drinking are modifiable factors influencing cardiovascular risk. Leukocyte count has been shown to be associated with smoking and alcohol drinking: Cigarette smoking causes an elevation of leukocyte count, and smoking cessation leads to recovery of leukocyte count [5–7]. Leukocyte count was shown to be inversely associated with alcohol consumption [6, 8]. Thus, smoking and drinking are thought to show opposite effects on leukocyte count.

Lipid-related indices, consisting of two or more variables including blood lipids, have been proposed as a discriminator of cardiometabolic risk. The ratio of LDL cholesterol to HDL cholesterol (LDL-C/HDL-C ratio) is a classical atherogenic index that predicts eventual coronary heart disease [9]. The ratio of triglycerides to HDL cholesterol (TG/HDL-C ratio) has been proposed as a better predictor than LDL-C/HDL-C ratio for cardiovascular disease [10, 11] and has been shown to reflect small dense LDL [12], a potent risk factor of cardiovascular diseases that is independent of LDL cholesterol levels [13]. More recently, cardiometabolic index (CMI), which is calculated as the product of TG/HDL-C ratio and the ratio of waist circumference to height (waist-to-height ratio), has been proposed as a discriminator of diabetes [14] and has been shown to be associated with atherosclerosis in patients with peripheral arterial disease [15] and to discriminate the risk of incidental coronary heart disease [16]. TG/HDL-C ratio has been reported to be correlated positively with leukocyte count in overweight and obese adolescents [17]. However, there has been little information on the relationships of leukocyte count with lipid-related indices in adults. Moreover, it remains to be determined whether and how these relationships are confounded by smoking and alcohol drinking.

The aim of this study was therefore to clarify whether and how smoking and alcohol drinking affect the relationships between leukocyte count and lipid-related indices. The relationship between leukocyte count and metabolic syndrome, a cluster of accumulated cardiovascular risks, including obesity, dyslipidemia, hypertension and diabetes [18, 19], was also investigated with adjustment for smoking and alcohol drinking. In addition, the above relationships between leukocyte count and cardiometabolic risk were investigated in different age groups of middle-aged men.

## Methods

### Subjects

The subjects were 11261 middle-aged men (30 ~ 65 years) who had undergone annual health checkup examinations at their workplaces in Yamagata Prefecture in Japan. The participants were working at various kinds of companies including construction, manufacturing, information and communications, transport, wholesale and retail trade, eating and drinking places, accommodations, and services. The participants were recruited from April 2008 to March 2009. Individuals with histories of infectious diseases, autoimmune diseases and malignant diseases that are known to influence leukocyte count in peripheral blood were excluded from the subjects of this study. The design and protocol of this study were approved by the Ethics Committee of Yamagata University School of Medicine (No. 112 in the period from April 2005 to March 2006) and the Hyogo College of Medicine Ethics Committee (No. 3003 in 2020).

Present histories of lifestyles, including smoking, alcohol drinking, regular exercise, illness and therapy for illness, were surveyed by using self-written questionnaires. Cigarette smokers were defined as those who had had a habit of smoking for 6 months or longer and had it for the past month or longer. The subjects were divided by average daily cigarette consumption into three groups of nonsmokers, light smokers (20 or less cigarettes per day) and heavy smokers (21 or more cigarettes per day). The subjects were also divided by frequency of weekly alcohol drinking into three groups of nondrinkers, occasional drinkers and regular drinkers, who answered as “never”, “sometimes” and “every day”, respectively, to the question, “How frequently do you drink alcohol?”. Regarding a habit of regular exercise, subjects who answered in the questionnaire that they perform exercise almost every day for 30 min or longer per day were defined as those with a habit of regular exercise. The subjects were divided into two groups of those with and those without a habit of regular exercise.

### Measurements

Height and body weight were measured with light clothes, and body mass index (BMI) was calculated as weight in kilograms divided by the square of height in meters. Waist circumference was measured at the navel level as defined by the Japanese Committee for the Diagnostic Criteria of Metabolic Syndrome [18]. Blood pressure was measured using a mercury sphygmomanometer after each subject had rested quietly in a sitting position with Korotkoff phase V being used to define diastolic pressure. Mean arterial pressure was defined as diastolic pressure plus one third of the difference between systolic pressure and diastolic pressure.

After overnight fasting, blood was collected from each subject in the morning. A part of the blood was immediately transferred to a 2-ml glass tube containing 3.8 mg EDTA-2K, and leukocyte count was measured by flow cytometry (with a red laser at 633 nm) using an automatic hematology analyzer (Sysmex XE-2100, Sysmex Corp., Kobe, Japan). The rest of the blood was used to obtain serum by centrifugation. Concentrations of triglycerides, HDL cholesterol and LDL cholesterol in serum were enzymatically measured with commercial kits by using an automatic analyzer (Hitachi Model 7350, Hitachi Corp., Tokyo, Japan). Hemoglobin A_1c_ was measured by the latex cohesion method using a commercial kit (Determiner HbA_1c_, Kyowa Medex, Tokyo, Japan). Hemoglobin A_1c_ values were calibrated by using a formula proposed by the Japan Diabetes Society as described before [20]. CMI was defined as the product of waist circumference (cm)-to-height (cm) ratio and triglycerides (mg/dl)-to-HDL cholesterol (mg/dl) ratio [14].

According to the criteria by the International Diabetes Federation [19] with a slight modification, metabolic syndrome was defined as the presence of two or more risk factors in addition to abdominal obesity diagnosed as high waist-to-height ratio. The risk factors included in the criteria of metabolic syndrome are abdominal obesity (high waist-to-height ratio), hypertension, dyslipidemia (high triglycerides and/or low HDL cholesterol) and diabetes evaluated by hemoglobin A_1c_ level and a history of medication therapy for diabetes. The criterion for each of the above risk factors was defined as follows: abdominal obesity, waist-to-height ratio ≥ 0.5 [21]; hypertension, systolic blood pressure ≥ 140 mmHg and/or diastolic blood pressure ≥ 90 mmHg [22]; high triglycerides, triglycerides ≥ 150 mg/dl; low HDL cholesterol, HDL cholesterol < 40 mg/dl [19, 23]; diabetes, hemoglobin A_1c_ ≥ 6.5% [24]. The cut-off values used for high LDL-C/HDL-C ratio, high TG/HDL-C ratio and high CMI were 3.5, 2.967 and 0.8, respectively [14, 25]. Subjects who were receiving medication therapy for hypertension and diabetes were included in the groups of subjects with hypertension and diabetes, respectively.

### Statistical analysis

Leukocyte counts of the subjects were arranged in ascending order. Then the subjects were divided into three tertile groups of approximately equal sizes. The subjects were also divided into four age groups of subjects at ages of 30~39, 40~49, 50~59 and 60~65 years. Continuous variables, which are summarized as means with standard deviations or medians with interquartile ranges, were compared among the groups of smoking, alcohol drinking, or tertile for leukocyte count by using analysis of variance (ANOVA) followed by the Scheffé’s F test as a post-hoc test in the univariable analysis and by using analysis of covariance (ANCOVA) followed by Student’s t-test with Bonferroni’s multiplicity correction in the multivariable analysis. Since levels of triglycerides, TG/HDL-C ratio and CMI did not display normal distributions, they were compared non-parametrically by using the Kruskal-Wallis test followed by the Steel-Dwass test as a post-hoc test in the univariable analysis and were compared by using ANCOVA after logarithmic transformation with a base of 10 in the multivariable analysis. Pearson’s chi-square test was used to compare categorical variables, which are summarized as frequencies and percentages. Dichotomous variables, including high LDL-C/HDL-C ratio, high TG/HDL-C ratio, high CMI and metabolic syndrome, were compared by using logistic regression analysis. In univariable logistic regression analysis, crude odds ratios of each group pair were compared by using the Breslow-Day test. Odds ratios of an interaction term consisting of the tertile group of leukocyte count and the age group were also estimated in multivariable logistic regression analysis. In ANCOVA and multivariable logistic regression analysis, adjustments were performed for age, BMI, habits of smoking, alcohol drinking and regular exercise, and a history of medication therapy for dyslipidemia as appropriate. All probability (*p*) values are two-sided, and *p* values less than 0.05 were defined as significant. The statistical analyses were performed using a computer software program (IBM SPSS Statistics for Windows, Version 25.0. Armonk, NY: IBM Corp).

## Results

### Characteristics of overall subjects and the tertile groups of subjects classified by leukocyte count

Table 1 shows profiles of overall subjects and subjects in the tertile groups of leukocyte count. The percentage of smokers, BMI, waist-to-height ratio, systolic blood pressure, diastolic blood pressure, triglycerides, LDL cholesterol, LDL-C/HDL-C ratio, TG/HDL-C ratio, CMI, hemoglobin A_1c_ and the percentage of subjects with diabetes tended to be higher with an increase of the tertile for leukocyte count. HDL cholesterol tended to be lower with an increase of the tertile for leukocyte count. Age and the percentage of drinkers were slightly but significantly younger and lower, respectively, in the 3rd tertile group of leukocyte count than in the 1st tertile group.

**Table 1:** Characteristics of overall subjects and subjects in each tertile group of leukocyte count.

	Leukocyte count 1st tertile	2nd tertile	3rd tertile	Overall subjects
Number	3706	3817	3738	11261
Leukocyte count (/μl)	4809 ± 600	6319 ± 396**	8577 ± 1463**,††	6572 ± 1806
Age (years)	47.5 ± 9.2	47.1 ± 9.1	46.6 ± 8.8**	47.1 ± 9.0
Smokers (%)	38.5	54.5**	75.4**,††	56.2
Drinkers (%)	81.8	81.4	75.7**,††	79.6
Regular exercise (%)	14.1	11.6**	9.3**,††	11.7
BMI (kg/m^2^)	22.96 ± 2.98	23.57 ± 3.26**	24.00 ± 3.54**,††	23.51 ± 3.30
Waist circumference (cm)	81.6 ± 8.5	83.5 ± 8.9**	84.5 ± 9.3**,††	83.2 ± 9.0
WHtR	0.480 ± 0.050	0.491 ± 0.052**	0.498 ± 0.054**,††	0.490 ± 0.053
SBP (mmHg)	125.1 ± 15.6	126.5 ± 16.6**	127.6 ± 16.6**,††	126.4 ± 16.3
DBP (mmHg)	77.1 ± 11.2	77.9 ± 11.6*	78.3 ± 11.6**	77.8 ± 11.4
MAP (mmHg)	93.1 ± 11.9	94.1 ± 12.5**	94.8 ± 12.5**	94.0 ± 12.3
Hypertension (%)	26.8	30.0**	30.9**	29.2
Therapy for hypertension (%)	12.2	12.5	11.7	12.2
Triglycerides (mg/dl)	88 (60, 132)	106 (74, 162)**	120 (82, 187)**,††	104 (71, 160)
HDL cholesterol (mg/dl)	60.6 ± 15.3	56.6 ± 14.4**	53.1 ± 13.8**,††	56.8 ± 14.8
LDL cholesterol (mg/dl)	113.1 ± 29.2	118.3 ± 30.5**	123.3 ± 32.2**,††	118.3 ± 30.9
LDL-C/HDL-C	2.00 ± 0.76	2.24 ± 0.84**	2.50 ± 0.94**,††	2.25 ± 0.88
TG/HDL-C	1.49 (0.93, 2.48)	1.95 (1.20, 3.29)**	2.33 (1.43, 4.04)**,††	1.90 (1.15, 3.23)
CMI	0.71 (0.43, 1.23)	0.96 (0.57, 1.69)**	1.16 (0.68, 2.08)**,††	0.93 (0.54, 1.65)
Therapy for dyslipidemia (%)	4.7%	5.8%*	5.4%	5.3%
Hemoglobin A_1c_ (%)	5.34 ± 0.56	5.46 ± 0.69**	5.55 ± 0.80**,††	5.45 ± 0.69
Therapy for diabetes (%)	3.1%	4.2%*	4.7%**	4.0%
Diabetes (%)	4.4%	6.5%**	7.9%**,†	6.3%

Shown are numbers, percentages, means with standard deviations, and medians with interquartile ranges in parentheses. WHtR, waist-to-height ratio; SBP, systolic blood pressure; DBP, diastolic blood pressure; MAP, mean arterial pressure. Symbols indicate significant differences from the 1st tertile group (*, *p* < 0,05; **, *p* < 0.01) and 2nd tertile group (†, *p* < 0,05; ††, *p* < 0.01).

### Correlations of leukocyte count with cardiometabolic risk factors

S1 Table shows Spearman’s rank correlation coefficients between cardiovascular risk factors. Leukocyte count was significantly correlated with waist-to-height ratio, mean arterial pressure, triglycerides, HDL cholesterol, LDL cholesterol, LDL-C/HDL-C ratio, TG/HDL-C ratio, CMI and hemoglobin A_1c_. The correlation coefficients of leukocyte count with the lipid-related indices, LDL-C/HDL-C ratio (*r* = 0.247, *p* < 0.01), TG/HDL-C ratio (*r* = 0.256, *p* < 0.01) and CMI (*r* = 0.258, *p* < 0.01), tended to be higher than those with other cardiovascular risk factors. Mean arterial pressure and hemoglobin A_1c_ showed stronger correlations with waist-to-height ratio than those with other cardiovascular risk factors.

### Comparison of mean levels of leukocyte count and lipid-related indices in the groups classified by a habit of smoking

Both in univariable analysis and multivariable analysis, mean levels of leukocyte count, TG/HDL-C ratio and CMI were significantly higher in light and heavy smokers than in nonsmokers and were significantly higher in heavy smokers than in light smokers (Table 2). Mean levels of LDL-C/HDL-C ratio were also significantly higher in light and heavy smokers than in nonsmokers and tended to be higher with an increase of cigarette consumption (Table 2).

**Table 2:** Comparison of mean levels of leukocyte count and lipid-related indices in non-, light and heavy smokers in overall subjects.

	Nonsmokers	Light smokers	Heavy smokers
Univariable			
Leukocyte count	5912 (5872 ~ 5951)	6887 (6834 ~ 6941)**	7584 (7492 ~ 7676)**,††
LDL-C/HDL-C	2.19 (2.16 ~ 2.21)	2.28 (2.25 ~ 2.30)**	2.33 (2.29 ~ 2.37)**
Log (TG/HDL-C)	0.263 (0.254 ~ 0.272)	0.318 (0.308 ~ 0.328)**	0.355 (0.339 ~ 0.371)**,††
Log (CMI)	-0.045 (-0.055 ~ -0.035)	0.001 (-0.010 ~ 0.011)**	0.042 (0.025 ~ 0.059)**,††
Multivariable			
Leukocyte count	5886 (5839 ~ 5932)	6913 (6864 ~ 6962)**	7592 (7516 ~ 7669)**,††
LDL-C/HDL-C	2.16 (2.14 ~ 2.19)	2.30 (2.28 ~ 2.33)**	2.33 (2.29 ~ 2.36)**
Log (TG/HDL-C)	0.253 (0.244 ~ 0.261)	0.329 (0.320 ~ 0.339)**	0.355 (0.340 ~ 0.369)**,†
Log (CMI)	-0.060 (-0.068 ~ -0.051)	0.017 (0.008 ~ 0.026)**	0.042 (0.028 ~ 0.057)**,†

Shown are means with their 95% confidence intervals in parentheses of leukocyte count, LDL-C/HDL-C ratio, log-transformed TG/HDL-C ratio and log-transformed CMI of non-, light and heavy smokers. In multivariable analysis, age, habits of alcohol drinking and regular exercise, and a history of medication therapy for dyslipidemia were adjusted. In addition, BMI was adjusted in analysis of log(LDL-C/HDL-C ratio) and log(TG/HDL-C ratio). “**” indicates significant differences (**, *p* < 0.01) from nonsmokers (significant differences between light smokers and nonsmokers and between heavy smokers and nonsmokers); “†” or “††” indicates significant differences (†, *p* < 0.05; ††, *p* < 0.01) from light smokers (significant differences between heavy and light smokers).

### Comparison of mean levels of leukocyte count and lipid-related indices in the groups classified by a habit of alcohol drinking

Both in univariable analysis and multivariable analysis, mean levels of leukocyte count and LDL-C/HDL-C ratio were significantly lower in occasional and regular drinkers than in nondrinkers and tended to be lower with an increase in frequency of drinking (Table 3). In univariable analysis, TG/HDL-C ratio and CMI were significantly lower in occasional and regular drinkers than in nondrinkers and tended to be lower with an increase in frequency of drinking, while there were no significant differences in TG/HDL-C ratio and CMI of non-, occasional and regular drinkers in multivariable analysis (Table 3).

**Table 3:** Comparison of mean levels of leukocyte count and lipid-related indices in non-, occasional and regular drinkers in overall subjects.

	Nondrinkers	Occasional drinkers	Regular drinkers
Univariable			
Leukocyte count	6833 (6755 ~ 6911)	6529 (6473 ~ 6585)**	6487 (6438 ~ 6536)**
LDL-C/HDL-C	2.60 (2.56 ~ 2.63)	2.34 (2.31 ~ 2.36)**	2.02 (2.00 ~ 2.04)**,††
Log (TG/HDL-C)	0.319 (0.306 ~ 0.332)	0.304 (0.294 ~ 0.314)	0.288 (0.278 ~ 0.298)**
Log (CMI)	0.006 (-0.008 ~ 0.020)	-0.007 (-0.017 ~ 0.005)	-0.025 (-0.036 ~ -0.015)**,†
Multivariable			
Leukocyte count	6829 (6761 ~ 6898)	6581 (6528 ~ 6634)**	6450 (6403 ~ 6496)**,††
LDL-C/HDL-C	2.57 (2.54 ~ 2.60)	2.31 (2.28 ~ 2.33)**	2.05 (2.03 ~ 2.07) **,††
Log (TG/HDL-C)	0.310 (0.297 ~ 0.323)	0.293 (0.283 ~ 0.303)	0.301 (0.292 ~ 0.309)
Log (CMI)	-0.005 (-0.018 ~ 0.009)	-0.020 (-0.031 ~ -0.010)	-0.010 (-0.019 ~ -0.001)

Shown are means with their 95% confidence intervals in parentheses of leukocyte count, LDL-C/HDL-C ratio, log-transformed TG/HDL-C ratio and log-transformed CMI of non-, occasional and regular drinkers. In multivariable analysis, age, habits of smoking and regular exercise, and a history of medication therapy for dyslipidemia were adjusted. In addition, BMI was adjusted in analysis of log(LDL-C/HDL-C ratio) and log(TG/HDL-C ratio). “**” indicates significant differences (**, *p* < 0.01) from nondrinkers (significant differences between occasional drinkers and nondrinkers and between regular drinkers and nondrinkers); “†” or “††” indicates significant differences (†, *p* < 0.05; ††, *p* < 0.01) from occasional drinkers (significant differences between regular and occasional drinkers).

### Comparison of mean levels of lipid-related indices in the tertile groups of leukocyte count in overall subjects and subjects without habits of smoking and alcohol drinking

As shown in Table 4, both in overall subjects and subjects without habits of smoking and alcohol drinking, mean levels of LDL-C/HDL-C ratio, TG/HDL-C ratio and CMI were significantly higher in the 2nd and 3rd tertile groups of leukocyte count than in the 1st tertile group and tended to be higher with an increase of the tertile. The above findings were observed both in univariable analysis and multivariable analysis.

**Table 4:** Comparison of mean levels of each lipid-related index in the tertile groups of leukocyte count in overall subjects and subjects without habits of smoking and alcohol drinking.

	Leukocyte count 1st tertile	2nd tertile	3rd tertile
Overall subjects			
Univariable			
LDL-C/HDL-C	2.00 (1.97 ~ 2.02)	2.24 (2.21 ~ 2.27)**	2.50 (2.46 ~ 2.53)**,††
Log (TG/HDL-C)	0.197 (0.187 ~ 0.208)	0.312 (0.301 ~ 0.322)**	0.389 (0.379 ~ 0.400)**,††
Log (CMI)	-0.123 (-0.134 ~ -0.112)	0.001 (-0.011 ~ 0.012)**	0.084 (0.073 ~ 0.095)**,††
Multivariable			
LDL-C/HDL-C	2.08 (2.05 ~ 2.10)	2.25 (2.22 ~ 2.27)**	2.41 (2.39 ~ 2.44)**,††
Log (TG/HDL-C)	0.225 (0.215 ~ 0.236)	0.311 (0.301 ~ 0.320)**	0.363 (0.352 ~ 0.373)**,††
Log (CMI)	-0.089 (-0.099 ~ -0.079)	-0.002 (-0.012 ~ 0.008)**	0.052 (0.042 ~ 0.063)**,††
Nonsmokers, nondrinkers			
Univariable			
LDL-C/HDL-C	2.16 (2.09 ~ 2.24)	2.42 (2.34 ~ 2.50)**	2.61 (2.52 ~ 2.70)**,††
Log (TG/HDL-C)	0.160 (0.131 ~ 0.189)	0.277 (0.245 ~ 0.308)**	0.362 (0.327 ~ 0.397)**,††
Log (CMI)	-0.166 (-0.197 ~ -0.135)	-0.034 (-0.069 ~ 0.000)**	0.066 (0.028 ~ 0.103)**,††
Multivariable			
LDL-C/HDL-C	2.25 (2.17 ~ 2.33)	2.43 (2.36 ~ 2.51)**	2.51 (2.43 ~ 2.59)**
Log (TG/HDL-C)	0.187 (0.156 ~ 0.218)	0.280 (0.252 ~ 0.308)**	0.329 (0.298 ~ 0.360)**
Log (CMI)	-0.123 (-0.153 ~ -0.092)	-0.031 (-0.060 ~ -0.003)**	0.018 (-0.013 ~ 0.049)**

Shown are means with their 95% confidence intervals in parentheses of LDL-C/HDL-C ratio, log-transformed TG/HDL-C ratio and log-transformed CMI of the tertile groups of leukocyte count. In multivariable analysis, age, a habit of regular exercise and a history of medication therapy for dyslipidemia were adjusted. Habits of smoking and alcohol drinking were also adjusted in analysis of overall subjects. In addition, BMI was adjusted in analysis of log(LDL-C/HDL-C ratio) and log(TG/HDL-C ratio). “**” indicates significant differences (**, *p* < 0.01) from the 1st tertile group of leukocyte count (significant differences between the 2nd and 1st tertile groups and between the 3rd and 1st tertile groups); “††” indicates significant differences (††, *p* < 0.01) from the 2nd tertile group of leukocyte count (significant differences between the 3rd and 2nd tertile groups).

### Comparison of prevalences of high lipid-related indices and metabolic syndrome in overall subjects and subjects without habits of smoking and alcohol drinking

As shown in Table 5, both in overall subjects and subjects without habits of smoking and alcohol drinking, prevalences of high LDL-C/HDL-C ratio, high TG/HDL-C ratio, high CMI and metabolic syndrome were significantly higher in the 2nd and 3rd tertile groups of leukocyte count than in the 1st tertile group and tended to be higher with an increase of the tertile.

**Table 5:** Comparison of prevalences of each cardiometabolic risk factor in the tertile groups of leukocyte count in overall subjects and subjects without habits of smoking and alcohol drinking.

	Leukocyte count 1st tertile	2nd tertile	3rd tertile	All tertiles
Overall subjects				
High LDL-C/HDL-C	3.8%	8.0%**	14.4%**,††	8.7%
High TG/HDL-C	18.6%	28.9%**	38.6%**,††	28.8%
High CMI	13.7%	23.3%**	31.5%**,††	22.9%
Metabolic syndrome	7.4%	11.8%**	14.6%**,††	11.3%
Nonsmokers, nondrinkers				
High LDL-C/HDL-C	4.4%	10.0%**	17.2%**,††	10.5%
High TG/HDL-C	13.1%	25.1%**	38.3%**,††	25.4%
High CMI	8.2%	20.1%**	32.3%**,††	20.1%
Metabolic syndrome	5.5%	12.1%**	16.3%**	11.3%

Shown are percentages of subjects with each cardiometabolic risk factor (high LDL-C/HDL-C ratio, high TG/HDL-C ratio, high CMI and metabolic syndrome) in the tertile groups of leukocyte count. Symbols denote significant differences from the 1st tertile group (**, *p* < 0.01) and the 2nd tertile group (††, *p* < 0.01) of leukocyte count.

### Odds ratios of the 2nd and 3rd tertile groups of leukocyte count vs. the 1st tertile group for high lipid-related indices and metabolic syndrome in overall subjects and subjects without habits of smoking and alcohol drinking

As shown in Table 6, both in overall subjects and subjects without habits of smoking and alcohol drinking, odds ratios of the 2nd and 3rd tertile groups vs. the 1st tertile group of leukocyte count for high LDL-C/HDL-C ratio, high TG/HDL-C ratio, high CMI and metabolic syndrome were significantly high when compared with the reference level of the 1st tertile group. The above odds ratios tended to be higher in the subject group of nonsmokers and nondrinkers than in the overall subjects.

**Table 6:** Odds ratios for each cardiometabolic risk factor in the 2nd and 3rd tertile groups vs. the 1st tertile group of leukocyte count.

	Leukocyte count 1st tertile	2nd tertile	3rd tertile
Overall subjects			
Univariable			
High LDL-C/HDL-C	1.00: reference	2.23 (1.81 ~ 2.74)**	4.28 (3.53 ~ 5.19)**
High TG/HDL-C	1.00: reference	1.78 (1.60 ~ 1.98)**	2.75 (2.48 ~ 3.06)**
High CMI	1.00: reference	1.93 (1.71 ~ 2.17)**	2.91 (2.59 ~ 3.27)**
Metabolic syndrome	1.00: reference	1.67 (1.42 ~ 1.95)**	2.12 (1.82 ~ 2.47)**
Multivariable			
High LDL-C/HDL-C	1.00: reference	1.89 (1.52 ~ 2.34)**	2.89 (2.34 ~ 3.56)**
High TG/HDL-C	1.00: reference	1.56 (1.39 ~ 1.75)**	2.15 (1.90 ~ 2.42)**
High CMI	1.00: reference	1.67 (1.47 ~ 1.90)**	2.23 (1.95 ~ 2.55)**
Metabolic syndrome	1.00: reference	1.54 (1.29 ~ 1.84)**	2.03 (1.68 ~ 2.46)**
Nonsmokers, nondrinkers			
Univariable			
High LDL-C/HDL-C	1.00: reference	2.44 (1.32 ~ 4.51)**	4.55 (2.52 ~ 8.20)**
High TG/HDL-C	1.00: reference	2.22 (1.50 ~ 3.27)**	4.11 (2.80 ~ 6.02)**
High CMI	1.00: reference	2.82 (1.78 ~ 4.48)**	5.38 (3.43 ~ 8.43)**
Metabolic synrome	1.00: reference	2.36 (1.35 ~ 4.11)**	3.33 (1.93 ~ 5.74)**
Multivariable			
High LDL-C/HDL-C	1.00: reference	2.04 (1.07 ~ 3.88)*	3.17 (1.72 ~ 5.85)**
High TG/HDL-C	1.00: reference	2.02 (1.34 ~ 3.04)**	3.20 (2.14 ~ 4.78)**
High CMI	1.00: reference	2.68 (1.63 ~ 4.41)**	4.05 (2.52 ~ 6.52)**
Metabolic syndrome	1.00: reference	2.09 (1.09 ~ 4.02)*	1.94 (1.04 ~ 3.61)*

Shown are odds ratios with their 95% confidence intervals in parentheses for each cardiometabolic risk factor (high LDL-C/HDL-C ratio, high TG/HDL-C ratio, high CMI and metabolic syndrome) of the 2nd and 3rd tertile groups vs. the 1st tertile group of leukocyte count. In multivariable analysis, age and a habit of regular exercise were used as explanatory variables. Habits of smoking and alcohol drinking were also used as explanatory variables in analysis of overall subjects. In addition, BMI was added to the explanatory variables in analysis of high LDL-C/HDL-C ratio and high TG/HDL-C ratio, and a history of medication therapy for dyslipidemia was used in analysis of high LDL-C/HDL-C ratio, high TG/HDL-C ratio and high CMI. Asterisks indicate significant odds ratios compared with the reference level (*, *p* < 0.05; **, *p* < 0.01).

### Relationships between age and cardiovascular risk factors

Results of comparison of cardiovascular risk factors in the age groups (30 ~ 39, 40 ~ 49, 50 ~ 59 and 60 ~ 65 years) are shown in Table 7. Leukocyte count was significantly lower in the oldest group than in the youngest group and tended to be lower with an increase of age. Waist-to-height ratio, mean arterial pressure, hemoglobin A_1c_ and the percentage of subjects with metabolic syndrome were significantly lower in the youngest group than in the other three groups and tended to be higher with an increase of age. Triglycerides, LDL cholesterol, TG/HDL-C ratio, CMI and the percentages of subjects with high TG/HDL-C ratio and high CMI were higher in the 40 ~ 49 and 50 ~ 59 years groups than in the other two groups, thus showing inverted U-shaped relationships with age. HDL cholesterol was slightly but significantly higher in the 50 ~ 59 and 60 ~ 65 years groups than in the youngest group and tended to be higher with an increase of age.

**Table 7:** Comparison of cardiometabolic risk factors in the different age groups of overall subjects.

	30 ~ 39 years	40 ~ 49 years	50 ~ 59 years	60 ~ 65 years
Leukocyte count (/μl)	6640 ± 1842	6642 ± 1879	6513 ± 1746	6341 ± 1610**
BMI (kg/m^2^)	23.36 ± 3.63	23.68 ± 3.39**	23.47 ± 3.03	23.49 ± 2.85
WHtR	0.477 ± 0.055	0.489 ± 0.052**	0.496 ± 0.050**	0.506 ± 0.047**
MAP (mmHg)	90.0 ± 11.2	93.3 ± 12.2**	96.9 ± 12.3**	97.5 ± 12.0**
Triglycerides (mg/dl)	96 (66, 152)	107 (73, 164)**	108 (73, 163)**	98 (69, 155)
HDL cholesterol (mg/dl)	56.1 ± 14.3	56.3 ± 14.9	57.4 ± 14.9*	58.3 ± 15.7**
LDL cholesterol (mg/dl)	115.1 ± 30.8	120.7 ± 31.7**	118.8 ± 30.6**	116.4 ± 29.0
LDL-C/HDL-C ratio	2.21 ± 0.89	2.31 ± 0.90**	2.23 ± 0.85	2.15 ± 0.82
TG/HDL-C ratio	1.75 (1.10, 3.02)	2.00 (1.21, 3.40)**	1.97 (1.19, 3.27)**	1.74 (1.06, 3.04)
CMI	0.826 (0.496, 1.477)	0.988 (0.560, 1.714)**	0.983 (0.565, 1.699)**	0.870 (0.504, 1.588)
High LDL-C/HDL-C ratio (%)	8.4	10.1*	8.1	6.9
High TG/HDL-C ratio (%)	25.9	30.5**	30.1**	25.9
High CMI (%)	20.4	24.3**	23.7**	21.7
Hemoglobin A_1c_ (%)	5.27 ± 0.53	5.40 ± 0.68**	5.57 ± 0.74**	5.69 ± 0.79**
Metabolic syndrome (%)	4.9	9.1**	16.1**	19.7**

Shown are percentages, means with standard deviations and medians with interquartile ranges in parentheses. WHtR, waist-to-height ratio; MAP, mean arterial pressure. Asterisks indicate significant differences from the youngest group (*, *p* < 0,05; **, *p* < 0.01).

### Correlations between leukocyte count and lipid-related indices in the different age groups

In all of the age groups, leukocyte count was significantly correlated with the three lipid-related indices (S2 Table). The correlation coefficients with log-transformed TG/HDL-C ratio and CMI were lower in the oldest group than in the youngest group (TG/HDL-C ratio, 0.195 vs. 0.253 [*p* = 0.084]; CMI, 0.191 vs. 0.263 [*p* < 0.05]).

### Odds ratios of the 2nd and 3rd tertile groups vs. the 1st tertile group of leukocyte count for high lipid-related indices and metabolic syndrome in the different age groups

As shown in Table 8, odds ratios of the 2nd and 3rd tertile groups vs. the 1st tertile group of leukocyte count for high lipid-related indices and metabolic syndrome were significantly high when compared with the reference level in all of the age groups except for the odds ratio of the 2nd vs. 1st tertile groups for high LDL-C/HDL-C ratio in the group of 60~65 years. The odds ratios tended to be lower in the oldest group than in the youngest group. Odds ratios of the interaction term consisting of age group (each age group vs. the youngest group) and leukocyte group (the 3rd vs. 1st tertile groups) for high TG/HDL ratio, high CMI and metabolic syndrome were significantly low in the oldest group when compared with the reference level.

**Table 8:** Odds ratios for each cardiometabolic risk factor of the 2nd and 3rd tertile groups vs. the 1st tertile group of leukocyte count in different age groups of overall subjects.

	30~39 years	40~49 years	50~59 years	60~65 years
High LDL-C/HDL-C				
1st tertile of leukocyte count	1.00: reference	1.00: reference	1.00: reference	1.00: reference
2nd tertile of leukocyte count	1.82 (1.16~2.85)**	1.88 (1.33~2.65)**	2.30 (1.52~3.47)**	1.26 (0.63~2.51)
3rd tertile of leukocyte count	2.70 (1.76~4.14)**	2.75 (1.96~3.85)**	3.53 (2.35~5.32)**	2.32 (1.18~4.54)*
[Interaction term]	[1.00: reference]	[1.25 (0.87~1.79)]	[1.33 (0.81~2.17)]	[1.05 (0.52~2.11)]
High TG/HDL-C				
1st tertile of leukocyte count	1.00: reference	1.00: reference	1.00: reference	1.00: reference
2nd tertile of leukocyte count	1.92 (1.51~2.45)**	1.81 (1.50~2.19)**	1.66 (1.37~2.01)**	1.42 (1.01~1.99)*
3rd tertile of leukocyte count	2.86 (2.24~3.64)**	2.80 (2.30~3.40)**	2.21 (1.80~2.71)**	1.85 (1.29~2.65)**
[Interaction term]	[1.00: reference]	[0.96 (0.76~1.21)]	[0.77 (0.59~1.02)]	[0.60 (0.41~0.90)*]
High CMI				
1st tertile of leukocyte count	1.00: reference	1.00: reference	1.00: reference	1.00: reference
2nd tertile of leukocyte count	2.05 (1.57~2.68)**	1.92 (1.55~2.36)**	1.90 (1.54~2.36)**	1.60 (1.12~2.29)*
3rd tertile of leukocyte count	2.77 (2.12~3.63)**	3.07 (2.48~3.79)**	2.66 (2.12~3.33)**	1.73 (1.17~2.56)**
[Interaction term]	[1.00: reference]	[1.05 (0.82~1.34)]	[0.92 (0.68~1.24)]	[0.58 (0.38~0.90)*]
Metabolic syndrome				
1st tertile of leukocyte count	1.00: reference	1.00: reference	1.00: reference	1.00: reference
2nd tertile of leukocyte count	3.90 (2.12~7.17)**	1.85 (1.34~2.56)**	1.95 (1.58~2.41)**	1.44 (1.01~2.06)*
3rd tertile of leukocyte count	5.11 (2.79~9.38)**	2.83 (2.06~3.90)**	2.69 (2.08~3.48)**	1.73 (1.16~2.58)**
[Interaction term]	[1.00: reference]	[1.04 (0.82~1.33)]	[0.72 (0.44~1.16)]	[0.26 (0.14~0.51)**]

Shown are odds ratios with their 95% confidence intervals in parentheses for each cardiometabolic risk factor (high LDL-C/HDL-C ratio, high TG/HDL-C ratio, high CMI and metabolic syndrome) of the 2nd and 3rd tertile groups vs. the 1st tertile group of leukocyte count. Odds ratios of the interaction term consisting of age group (each age group vs. the youngest group) and tertile group of leukocyte count (the 3rd vs. 1st tertiles) were also estimated. Age and histories of smoking, alcohol drinking and regular exercise were adjusted. In addition, BMI was adjusted in analysis of high LDL-C/HDL-C ratio and high TG/HDL-C ratio, and a history of medication therapy for dyslipidemia was adjusted in analysis of high LDL-C/HDL-C ratio, high TG/HDL-C ratio and high CMI. Asterisks indicate significant odds ratios compared with the reference level (*, *p* < 0.05; **, *p* < 0.01).

## Discussion

Both in overall subjects and subjects not having habits of smoking and alcohol drinking, leukocyte count was demonstrated to be positively associated with three lipid-related indices, including HDL-C/LDL-C ratio, TG/HDL-C ratio and CMI, and metabolic syndrome in ANCOVA and logistic regression analysis. Therefore, leukocyte count is associated with lipid-related indices and metabolic syndrome independently of habits of smoking and alcohol drinking. Leukocyte count and lipid-related indices were significantly higher in smokers than in nonsmokers and tended to be higher with an increase of cigarette consumption. Thus, smoking is a confounder for the relationship between leukocyte count and lipid-related indices. Leukocyte count and LDL-C/HDL-C ratio were significantly lower in drinkers than in nondrinkers and tended to be lower with an increase in frequency of drinking, suggesting that drinking is also a confounder of the association between leukocyte count and LDL-C/HDL-C ratio. Nonetheless, the associations of leukocyte count with the lipid-related indices and metabolic syndrome were independent of smoking and alcohol drinking as shown in the analyses of nonsmokers and nondrinkers alone and the multivariable analyses with adjustment for smoking and drinking. The three lipid-related indices evaluated in this study consist of HDL cholesterol, LDL cholesterol, triglycerides, and waist-to-height ratio, which were associated with leukocyte count (Table 1). In addition to these variables, blood pressure and hemoglobin A_1c_, which were also associated with leukocyte count (Table 1), are included in the criteria of metabolic syndrome. Therefore, it is reasonable that leucocyte count was associated with the lipid-related indices and metabolic syndrome. Interestingly, each odds ratio for high lipid-related indices of the 2nd or 3rd tertiles vs. the 1st tertile for leukocyte count in nonsmokers and nondrinkers tended to be higher than the corresponding odds ratio in overall subjects (Table 6). This suggests that the associations between leukocyte count and lipid-related indices after excluding effects of smoking and alcohol drinking are stronger than the associations before excluding them. To the best of my knowledge, this is the first study showing the relationships of leukocyte count with lipid-related indices and metabolic syndrome in relation to habits of smoking and drinking.

Leukocyte count, lipid-related indices and metabolic syndrome were also influenced by age: Leukocyte count was significantly lower in the oldest group of 60 ~ 65 years than in the youngest group of 30 ~ 39 years and tended to be lower with an increase of age; LDL-C/HDL-C ratio was significantly higher in the group of 40 ~ 49 years than in the group of 30 ~ 39 years; TG/HDL-C ratio and CMI were significantly higher in the groups of 40 ~ 49 and 50 ~ 59 years than in the group of 30 ~ 39 years; the prevalence of metabolic syndrome tended to be higher with an increase of age (Table 7). Thus, age is also a confounder for the relationships of leukocyte count with the lipid-related indices and metabolic syndrome. Therefore, age was always included in the explanatory variables in the multivariable analyses. However, as shown in S2 Table and Table 8, leukocyte count was associated with LDL-C/HDL-C ratio, TG/HDL-C ratio, CMI and metabolic syndrome in all of the age groups. Thus, these associations are independent of age. Interestingly, the correlation coefficients of leukocyte count with log-transformed TG/HDL-C ratio and log-transformed CMI tended to be lower with an increase of age, and the odds ratios of the interaction term consisting of age (vs. the youngest group) and leukocyte tertile (vs. the 1st tertile) for high TG/HDL-C, high CMI and metabolic syndrome were significantly lower in the group of 60 ~ 65 years compared with the reference level (30 ~ 39 years), suggesting weaker associations of leukocyte count with high TG/HDL-C, high CMI and metabolic syndrome in the oldest group than in the youngest group (Table 8). Moreover, the odds ratios for high TG/HDL-C ratio, high CMI and metabolic syndrome of the 2nd and 3rd tertiles for leukocyte count vs. the 1st tertile tended to be lower with an increase of age. Therefore, the associations of leukocyte count with high TG/HDL-C ratio, high CMI and metabolic syndrome are suggested to be weaker with age in middle-aged men. Although the reason for this age-dependent difference is unknown, one possible reason is higher variability of leukocyte count levels due to higher frequency of latent inflammatory diseases in older men. However, standard deviations of leukocyte count were not higher in older groups than in younger groups (Table 7).

Since atherosclerosis includes an inflammatory process [26], it is reasonable that higher leukocyte count is associated with higher risk of cardiovascular disease and its risk factors including visceral obesity and dyslipidemia, which are components of lipid-related indices. In this study, the association between leukocyte count and cardiometabolic risk remained significant after adjustment for various confounding factors including age, BMI, smoking, alcohol drinking and regular exercise. This finding supports the hypothesis that a high leukocyte count in healthy individuals reflects an inflammatory process in the pathogenesis of atherosclerosis. A recent study in which a longitudinal analysis was performed using a database of 9058 participants showed a robust positive association between triglycerides and leukocyte count in peripheral blood [27]. The authors of that report suggested a direct involvement of triglycerides in leukogenesis. The risk of high lipid-related indices in subjects without habits of smoking and drinking were three- to four-fold higher in the 3rd tertile group of leukocyte count than in the 1st tertile group in multivariable logistic regression analysis (Table 6). Thus, leukocyte count, which is a simple measure and is usually included in general health checkup examinations, is thought to be a useful variable to evaluate cardiovascular risk, although leukocyte count is influenced by infection and various inflammatory diseases. Because healthy individuals with a high leukocyte count in repeated health checkup examinations are suspected to have considerable atherosclerotic progression, they should be recommended to receive further examinations of atherosclerosis including ultrasonography of carotid arteries and aortic pulse wave velocity. The findings of stronger associations of leukocyte count with lipid-related indices and metabolic syndrome in younger subjects suggest that leukocyte count is more useful for discriminating cardiometabolic risk in younger middle-aged men than in older men.

### Strengths

This study clearly demonstrated that leukocyte count in peripheral blood shows associations with cardiometabolic risk evaluated by prevalence of metabolic syndrome and high levels of lipid-related indices. These associations were independent of age and habits of smoking and alcohol drinking, although they confounded the relationships between leukocyte count and cardiometabolic risk. Thus, leukocyte count is a simple useful marker for discriminating cardiometabolic risk. It was also found in this study that the associations between leukocyte count and cardiometabolic risk declined with an increase of age (30 ~ 65 years). This suggests that leukocyte count is more useful as a discriminator of cardiometabolic risk in younger men.

### Limitations

The subjects were middle-aged Japanese men. An age-dependent significant decline in the strength of the associations of leukocyte count with lipid-related indices and metabolic syndrome was observed in the group of 60 ~ 65 years, which is the oldest category in general workplaces. Thus, the above associations need to be investigated in further older subjects. Both the profiles of blood lipids and leukocyte count have been reported to differ by gender [28, 29]. It is known that there are ethnic differences in blood lipid levels and the risk of cardiovascular diseases [30]. Therefore, further studies using data for female subjects and subjects with other ethnicities are also needed to confirm the findings of the present study. The subjects were workers who had undergone annual health checkups at various companies, and thus there is a selection bias because they were generally healthier than those not receiving annual health checkups. Drinking, smoking and regular exercise were self-reported and thus there are possibilities of biases caused by underreporting or overreporting of them. Regarding the definition of smokers, subjects who had a habit of smoking for the past month or longer were categorized as smokers. However, there is a possibility that effects of cigarette smoking on leukocyte count and cardiometabolic risk persisted in subjects who recently quit smoking. The participants were recruited from April 2008 to March 2009, and at that time, individuals with a habit of vaping were rare in Japan. In a large population-based follow-up study in the UK, a positive association between leukocyte count and cardiovascular events mainly depended on the increase in neutrophils [31]. In a population-based cross-sectional study in the US, lymphocyte and basophil counts showed relatively strong positive and inverse correlations with triglycerides and HDL cholesterol, respectively [32]. However, information on leukocyte subtypes was not available in the present study. Since this study is cross-sectional in its design, further prospective studies are needed to discuss causal relationships of leukocyte count with lipid-related indices and metabolic syndrome.

## Conclusion

There are clear associations of lipid-related indices and metabolic syndrome with total leukocyte count in middle-aged Japanese men, and the associations are independent of alcohol consumption and cigarette smoking. Leukocyte count in peripheral blood, a simple measure usually included in general health checkup examinations, is a more useful discriminator of cardiometabolic risk in young middle-aged men than in older men.

## Supporting information

S1 Table Correlations between each pair of leukocyte count and cardiometabolic risk factors in overall subjects. Shown are Spearman’s rank correlation coefficients between each pair of leukocyte count and cardiometabolic risk factors in overall subjects. WHtR, waist-to-height ratio; MAP, mean arterial pressure. Asterisks denote significant correlations (**, *p* < 0.01).
(DOCX)

S2 Table Correlations between leukocyte count and each lipid-related index in different age groups of overall subjects. Shown are Pearson’s correlation coefficients between leukocyte count and each lipid-related index (LDL-C/HDL-C ratio, TG/HDL-C ratio and CMI). Symbols indicate significant (**, *p* < 0.01) correlation coefficients and significant (†, *p* < 0.05) or marginally significant (#, *p* = 0.084) differences from the corresponding correlation coefficients in the youngest (30~39 years) group.
(DOCX)

S1 Dataset (XLSX)
