# Supplementary material for: Associations between leukocyte count and lipid-related indices: Effect of age and confounding by habits of smoking and alcohol drinking
Source: PLoS One. 2023 Jan 31;18(1):e0281185. doi: 10.1371/journal.pone.0281185 (PMC9888682; doi:10.1371/journal.pone.0281185)
Supplement: S1 Table — Shown are Spearman’s rank correlation coefficients between each pair of leukocyte count and cardiometabolic risk factors in overall subjects. WHtR, waist-to-height ratio; MAP, mean arterial pressure. Asterisks denote significant correlations (**, p < 0.01). (DOCX) [file pone.0281185.s001.docx]

**S1 Table.** Correlations between each pair of leukocyte count and cardiometabolic risk factors in overall subjects.

|  | Leukocyte count | WHtR | MAP | Tri-glycerides | HDL-C | LDL-C | LDL-C/HDL-C | TG/HDL-C | CMI | Hemo-globin A_1c_ |
| --- | --- | --- | --- | --- | --- | --- | --- | --- | --- | --- |
| Leukocyte count | 1.000** |  |  |  |  |  |  |  |  |  |
| WHtR | 0.148** | 1.000 |  |  |  |  |  |  |  |  |
| MAP | 0.063** | 0.370** | 1.000 |  |  |  |  |  |  |  |
| Triglycerides | 0.224** | 0.361** | 0.197** | 1.000 |  |  |  |  |  |  |
| HDL-C | -0.230** | -0.341** | -0.018 | -0.469** | 1.000 |  |  |  |  |  |
| LDL-C | 0.145** | 0.288** | 0.094** | 0.217** | -0.188** | 1.000 |  |  |  |  |
| LDL-C/HDL-C | 0.247** | 0.407** | 0.074** | 0.437** | -0.745** | 0.768** | 1.000 |  |  |  |
| TG/HDL-C | 0.256** | 0.401** | 0.163** | 0.951** | -0.704** | 0.240** | 0.599** | 1.000 |  |  |
| CMI | 0.258** | 0.506** | 0.203** | 0.940** | -0.705** | 0.261** | 0.614** | 0.991** | 1.000 |  |
| Hemoglobin A_1c_ | 0.159** | 0.303** | 0.144** | 0.165** | -0.150** | 0.208** | 0.234** | 0.181** | 0.210** | 1.000 |

Shown are Spearman’s rank correlation coefficients between each pair of leukocyte count and cardiometabolic risk factors in overall subjects. WHtR, waist-to-height ratio; MAP, mean arterial pressure. Asterisks denote significant correlations (**, *p* < 0.01).
