# Supplementary material for: Associations between leukocyte count and lipid-related indices: Effect of age and confounding by habits of smoking and alcohol drinking
Source: PLoS One. 2023 Jan 31;18(1):e0281185. doi: 10.1371/journal.pone.0281185 (PMC9888682; doi:10.1371/journal.pone.0281185)
Supplement: S2 Table — Shown are Pearson’s correlation coefficients between leukocyte count and each lipid-related index (LDL-C/HDL-C ratio, TG/HDL-C ratio and CMI). Symbols indicate significant (**, p < 0.01) correlation coefficients and significant (†, p < 0.05) or marginally significant (#, p = 0.084) differences from the corresponding correlation coefficients in the youngest (30~39 years) group. (DOCX) [file pone.0281185.s002.docx]

**S2 Table.** Correlations between leukocyte count and each lipid-related index in different age groups of overall subjects.

|  | 30~39 years  (n = 2814) | 40~49 years  (n = 3823) | 50~59 years  (n = 3499) | 60~65 years  (n = 1125) |
| --- | --- | --- | --- | --- |
| LDL-C/HDL-C  Log(TG/HDL-C)  Log(CMI) | 0.239**  0.253**  0.263** | 0.253**  0.246**  0.253** | 0.247**  0.211**  0.215** | 0.194**  0.195**,#  0.191**,† |

Shown are Pearson’s correlation coefficients between leukocyte count and each lipid-related index (LDL-C/HDL-C ratio, TG/HDL-C ratio, and CMI). Symbols indicate significant (**, *p* < 0.01) correlation coefficients and significant (†, *p* < 0.05) or marginally significant (#, *p* = 0.084) differences from the corresponding correlation coefficients in the youngest (30~39 years) group.
